# Cost‐Effectiveness Analysis of Ritlecitinib Compared With No Treatment in Patients With Severe Alopecia Areata in Japan

**DOI:** 10.1111/1346-8138.70212

**Published:** 2026-03-09

**Authors:** Akira Yuasa, Kazumasa Kamei, Shota Saito, Masashi Mikami, Tatsunori Murata, Samantha Kiyomi Kurosky, Ernest H. Law, Kouki Nakamura, Rie Ueki

**Affiliations:** ^1^ Japan Access and Value, Pfizer Japan Inc. Tokyo Japan; ^2^ Health Economic Research Department CRECON Medical Assessment Inc. Tokyo Japan; ^3^ HTA, Value and Evidence, Pfizer Inc. New York New York USA; ^4^ Medical Affairs, Pfizer Japan Inc. Tokyo Japan; ^5^ Department of Dermatology Juntendo Tokyo Koto Geriatric Medical Center Tokyo Japan

**Keywords:** alopecia areata, cost‐effectiveness analysis, employer health costs, Japan, ritlecitinib

## Abstract

The efficacy and safety of ritlecitinib, a dual inhibitor of Janus kinase 3 (JAK3) and tyrosine kinase expressed in hepatocellular carcinoma (TEC) family kinases, have been demonstrated in the ALLEGRO phase 2b/3 trial that enrolled patients aged ≥ 12 years with alopecia areata (AA) and ≥ 50% scalp hair loss. The present analysis evaluated the cost‐effectiveness of ritlecitinib compared with no treatment for patients with AA and ≥ 50% scalp hair loss in Japan from a societal perspective. A Markov model stratified by health states based on the Severity of Alopecia Tool (SALT) was developed to estimate the lifetime costs, including productivity loss, and quality‐adjusted life years (QALYs) associated with the treatment of AA. The comparator was no treatment, and clinical parameters were based on the ALLEGRO phase 2b/3 trial. Costs and QALYs were discounted at an annual rate of 2%, and the incremental cost‐effectiveness ratio (ICER) was calculated. Over a lifetime horizon and with the base‐case societal perspective, the incremental QALYs and incremental costs of ritlecitinib 50 mg were 1.09 QALYs and 5 262 471 Japanese yen (JPY) (34 766 United States dollars [USD]), respectively. The ICER was 4 816 589 JPY (31 820 USD) per QALY, which was below the cost‐effectiveness threshold in Japan of 5 000 000 JPY (33 032 USD) per QALY. A one‐way sensitivity analysis showed that parameters with the most notable effect on the ICER were the utility for patients with SALT score ≥ 50, percentage of work time missed due to presenteeism for patients with SALT score ≥ 50, and the discontinuation rate of ritlecitinib 50 mg. These results indicate that ritlecitinib 50 mg is cost‐effective compared with no treatment for patients with AA and hair loss on ≥ 50% of the scalp from a societal perspective in Japan.

## 1 Introduction

Alopecia areata (AA) is an autoimmune disease caused by immune privilege collapse at hair follicles, followed by autoreactive T cell attacks and sometimes becomes chronic with recurrent episodes [1]. AA affects both males and females of all ages and ethnicities. AA tends to be underestimated or even dismissed as simply a cosmetic problem; however, especially in severe cases, the disease not only impairs the patients' body image but may also affect their quality of life [2, 3].

The lifetime incidence of AA has been estimated as approximately 0.58%–2% worldwide [4, 5, 6, 7, 8]. In addition, as per a claims database study, the prevalence of AA in Japan was 0.16% in 2012 and 0.27% in 2019, with a gradual increasing trend thereafter [9]. A web‐based survey of the general population in Japan reported that the prevalence of AA was 1.45% [10], which suggests that many patients with AA are not receiving treatment from a specialist.

Increased levels of hair loss tend to reduce the health‐related quality of life (HRQoL) of patients owing to increased disruption in daily life and difficulties in work or academic activities [11]. Patients with AA often experience symptoms of depression and require psychological care as well as appropriate treatment for hair loss [12]. A recent study in Japan reported that AA results in > 2 million days of lost activity per year and that the estimated cost associated with productivity loss is 88.1 billion Japanese yen (JPY) (745 million United States dollars [USD]) [13]. These results indicate that AA has a notable effect on both time and economic burden at the national level in Japan.

Ritlecitinib is an orally administered small‐molecule agent that irreversibly inhibits Janus kinase (JAK) 3 and tyrosine kinases expressed in hepatocellular carcinoma family kinases [14]. The ALLEGRO phase 2b/3 trial was a placebo‐controlled, randomized, double‐blind, parallel‐group comparative study conducted in 18 countries or regions, including Japan. At Week 24, the primary endpoint of the proportion of patients achieving a Severity of Alopecia Tool (SALT) score ≤ 20 was significantly higher in the ritlecitinib group (excluding the 10 mg dose) than the placebo group (placebo: 1.5% vs. ritlecitinib 50 mg: 23.4%, *p* < 0.001) [15].

In Japan, ritlecitinib was approved in 2023 for the treatment of severe AA (limited to intractable cases involving widespread hair loss) in adults and adolescents aged ≥ 12 years [16]. Most of these eligible patients have experienced conventional treatments (e.g., topical corticosteroids) but have not achieved sufficient therapeutic efficiency with them. However, other treatment options were limited for these patients, and they need to continue insufficient treatment or discontinue treatment for AA before the launch of ritlecitinib. Two double‐blind, randomized controlled trials in France and Iran have reported on the efficacy of oral methotrexate in patients with refractory or treatment‐resistant AA [17, 18]. In Japan, however, methotrexate is not an established treatment for patients with AA, with no coverage by health insurance or recommendation by clinical guidelines [3]. Therefore, a cost‐effectiveness analysis is needed for ritlecitinib in patients with ≥ 50% scalp hair loss compared with no treatment.

Here, we conducted a cost‐effectiveness analysis of ritlecitinib for patients with AA in Japan from a societal perspective.

## 2 Methods

### 2.1 Patient Population, Perspective, Intervention, and Comparator

According to the indication for ritlecitinib in Japan and the eligible patients in the ALLEGRO phase 2b/3 trial, the inclusion criteria for the current model were patients with ≥ 50% scalp hair loss, no signs of terminal hair regrowth within the past 6 months, and disease duration ≤ 10 years [19].

The comparator was no treatment. The latest clinical guidelines for AA in Japan indicate that JAK inhibitors are the only recommended treatment having evidence level A for patients with severe AA, where ritlecitinib is the only available JAK inhibitor for patients with AA aged 12 to 14 years old. We therefore consider the setting of the comparator as no treatment to be appropriate for patients with AA in Japan [3].

The base‐case analysis was conducted from a societal perspective in Japan, considering the costs associated with productivity loss related to AA.

### 2.2 Model Structure and Settings

**FIGURE 1:**
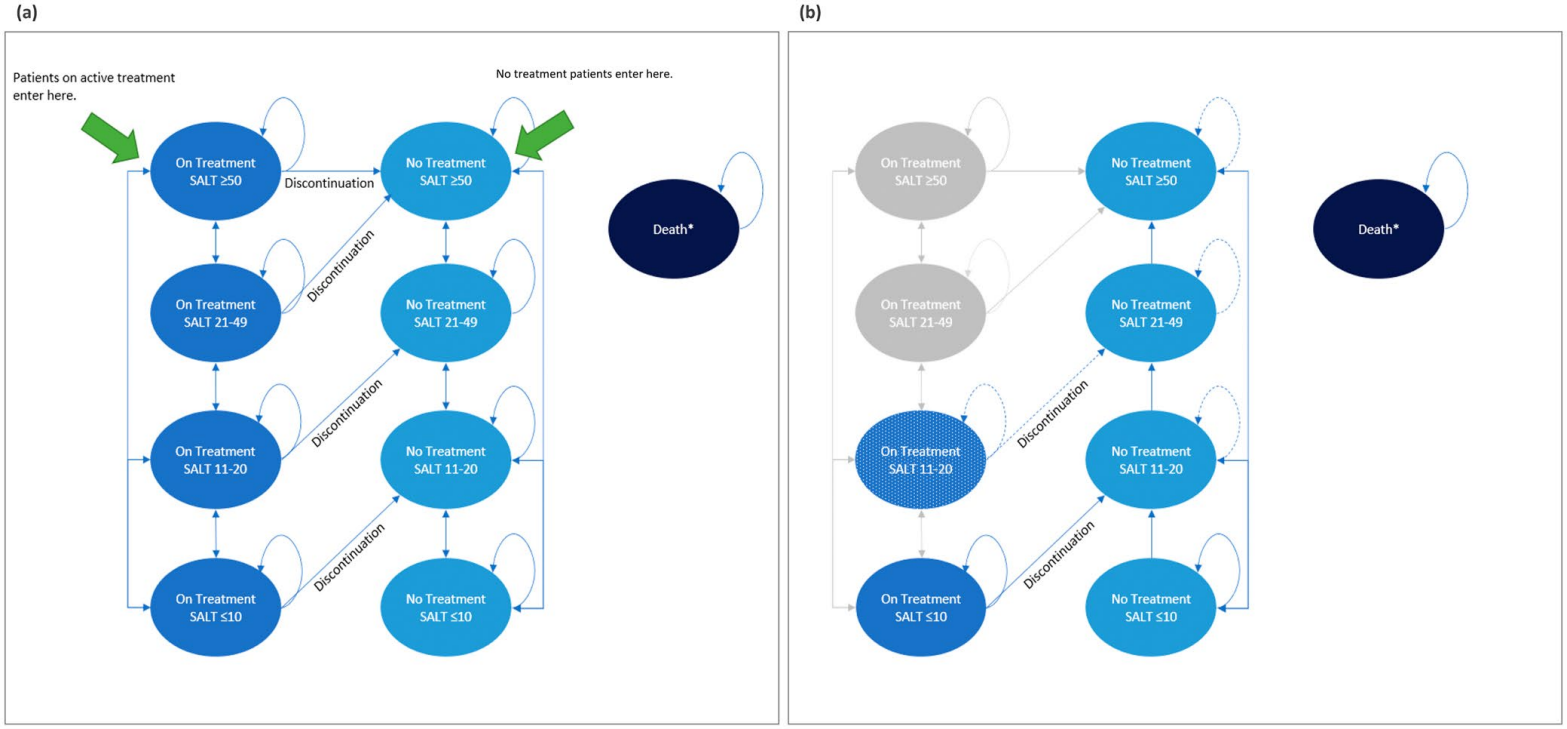
Model structure diagram of (a) Markov model up to Week 48, and (b) Markov model after Week 48. SALT: Severity of Alopecia Tool.

The Markov model was developed using Microsoft Excel (Microsoft, Redmond, WA, USA). The health states of patients with AA were stratified by SALT scores and the presence or absence of active treatment with ritlecitinib 50 mg (Figure 1) [20]. The SALT score ranges from 0 (no hair loss) to 100 (complete scalp hair loss) [21]. Patients receiving treatment with ritlecitinib 50 mg initiated the analysis as “active treatment (SALT score ≥ 50)” and those not receiving active treatment initiated as “no treatment (SALT score ≥ 50).” Subsequently, the model simulated the time‐dependent changes in SALT scores and the long‐term progression of treatment discontinuation with ritlecitinib 50 mg as per the efficacy observed in the ALLEGRO phase 2b/3 trial. The model also incorporated transitions to death from all health states.

In the ritlecitinib group, patients received ritlecitinib 50 mg to Week 48 (as per the secondary endpoint of the ALLEGRO phase 2b/3 trial), and those who did not achieve SALT score ≤ 20 at Week 48 were to discontinue treatment with ritlecitinib. Patients who achieved SALT score ≤ 20 at Week 48 continued ritlecitinib 50 mg after Week 48; however, discontinuation was assumed to occur in the ritlecitinib group in each cycle after Week 48. The discontinuation rate for ritlecitinib 50 mg (13.1% at Week 48) was calculated as per the treatment continuation rate at Week 48 in the ALLEGRO phase 2b/3 trial (Table 1). In cases where treatment with ritlecitinib 50 mg was discontinued, patients transitioned to a health state with a one‐step worsening in their SALT score.

**TABLE 1:** Key parameters for base‐case model.

Parameters	Inputs				References
Patient background					
Mean age	40.2 years				JMDC claims data
Percentage female	61.9%				
Population aged ≥ 12 to < 18 years	7.4%				
Clinical					
Treatment adherence of ritlecitinib 50 mg	97.4%				Ritlecitinib CSR [22]
SALT score distribution					
Ritlecitinib 50 mg	Week 12	Week 24	Week 34	Week 48	
SALT score ≥ 50	83.3%	57.3%	41.3%	36.4%	Ritlecitinib CSR [22]
SALT score 21–49	10.3%	19.4%	20.2%	14.5%	
SALT score 11–20	0.8%	9.7%	11.9%	13.6%	
SALT score ≤ 10	5.6%	13.7%	26.6%	35.5%	
No Treatment	Week 12	Week 24	Week 34	Week 48	
SALT score ≥ 50	97.6%	91.5%	—	—	Ritlecitinib CSR [22]
SALT score 21–49	0.8%	6.9%	—	—	
SALT score 11–20	0.8%	0.0%	—	—	
SALT score ≤ 10	0.8%	1.5%	—	—	
Treatment discontinuation of ritlecitinib 50 mg at Week 48	13.1%				ALLEGRO phase 2b/3 trial [15]
Adverse event incidence	Ritlecitinib 50 mg at Week 48		No treatment at Week 24		
Acne	9.2%		4.6%		ALLEGRO phase 2b/3 trial [15]
Constipation	0.8%		0.0%		
Diarrhea	9.2%		3.8%		
Dizziness	3.1%		0.8%		
Folliculitis	6.2%		2.3%		
Headache	12.3%		8.4%		
Nasopharyngitis	13.8%		6.1%		
Nausea	2.3%		5.3%		
Rash	5.4%		0.0%		
Upper respiratory tract infection	8.5%		7.6%		
Urticaria	5.4%		2.3%		
Utility weight					
SALT score ≥ 50	0.554				Aggio et al. (2024) [23]
SALT score 21–49	0.703				
SALT score 11–20	0.853				
SALT score ≤ 10	0.919				
Direct costs					
Drug acquisition cost (ritlecitinib; JPY)	5584.30 per 50 mg				NHI drug price list (revised in 2025) [24]
HCRU costs per month (JPY)	Drug cost		Non‐drug cost		
SALT score ≥ 50	3233		13 783		JMDC claims data
SALT score 21–49	3082		7967		
SALT score 11–20	3082		7967		
SALT score ≤ 10	1558		7730		
Adverse event management cost per event (JPY)					
Acne	625				Expert opinion
Constipation	25				
Diarrhea	84				
Dizziness	0				
Folliculitis	505				
Headache	68				
Nasopharyngitis	43				
Nausea	3				
Rash	21				
Upper respiratory tract infection	16				
Urticaria	237				
Indirect costs					
AA‐related productivity loss					
Percentage of AA population employed	83.3%				Derived from Japanese government statistics [25, 26]
Average work hours per week	36.8				Derived from Japanese government statistics [25, 26]
Average hourly wage (JPY)	2353				Derived from Japanese government statistics [25, 26]
Percentage of work time missed	Absenteeism		Presenteeism		
SALT score ≥ 50	1.5%		31.4%		Ohyama et al. (2023) [13]
SALT score 21–49	0.5%		30.0%		
SALT score 11–20	0.5%		13.9%		
SALT score ≤ 10	0.5%		13.9%		
Annual discount rate for cost and effectiveness	2%				Guideline for cost‐effectiveness evaluation in Japan [27]

Abbreviations: AA, alopecia areata; CSR, clinical study report; HCRU, healthcare resource utilization; JPY, Japanese yen; NHI; Japanese National Health Insurance; SALT, Severity of Alopecia Tool.

In the no‐treatment group, patients who achieved a SALT score ≤ 10 at Week 24 (primary endpoint of the ALLEGRO phase 2b/3 trial) were considered to have experienced spontaneous remission, without worsening in SALT score after Week 24. Patients who achieved a SALT score of 11–20 or 21–49 at Week 24 were considered to have experienced a gradual worsening of their SALT score over time.

According to the guideline for cost‐effectiveness analysis in Japan, we set model settings (time horizon, effectiveness measure, discount rate) [27]. The time horizon of the analysis was set to a lifetime because the guideline recommends that the time horizon should be sufficiently long. The cycle length was set at 12 weeks, and a half‐cycle correction was applied. The effectiveness measure was the quality‐adjusted life year (QALY), and the costs and QALYs estimated in the model were discounted at an annual rate of 2% [27].

The cost‐effectiveness analysis via a simulation model utilized data from previous trials, including the ALLEGRO phase 2b/3 trial, and information from published literature. The efficacy and safety data from the placebo arm in the ALLEGRO phase 2b/3 trial were extrapolated for the no‐treatment group in the model.

The willingness‐to‐pay (WTP) threshold in this analysis was set at 5000000 JPY (33 032 USD), as per the lowest reference value used in the Health Technology Assessment of Japan [28]. The threshold of 5 000 000 JPY/QALY used in Japan was established as a matter of policy, considering the results of a survey by the National Institute of Public Health in Japan on the willingness to pay of patients, alongside other relevant factors [27, 28]. The key parameters used in the Markov model are summarized in Table 1.

### 2.3 Effectiveness and Safety

Efficacy and safety inputs for the model were based on data from the ALLEGRO phase 2b/3 trial [15]. Data from the ritlecitinib 50 mg and placebo groups of the ALLEGRO phase 2b/3 trial were used for the ritlecitinib and no treatment groups of the Markov model, respectively.

Adverse events that occurred in ≥ 5% of patients in the ALLEGRO phase 2b/3 trial were incorporated into the analysis model. Excess mortality due to AA was not considered, given the absence of a direct association. In addition, the same general mortality rate was assumed between the ritlecitinib and no treatment groups as per the Abridged Life Tables for Japan from the Ministry of Health, Labour and Welfare [29].

### 2.4 Utility

The utility values for health states stratified by SALT score were derived from published literature. Aggio et al. estimated utility weights for SALT score‐based health states using the Time Trade‐Off (TTO) method, as per standardized patient‐reported outcome vignettes, in members of the general public (*n* = 120) in the UK [23].

In the ALLEGRO phase 2b/3 trial, the incidence of serious adverse events was low in the ritlecitinib 50 mg group. Disutility due to adverse events was therefore not considered in this model as their effect on HRQoL was considered limited.

### 2.5 Costs

The cost of ritlecitinib 50 mg and adverse event management were referenced as of April 2025. Healthcare resource utilization (HCRU) costs were calculated using the Medical Service Fee Points in the Japanese National Health Insurance (NHI) data as of June 2024, adjusted by average revision rates to reflect current value.

JPY was converted to USD using the 2024 exchange rate of the Organization for Economic Co‐operation and Development at 1 USD = 151.37 JPY [30].

Healthcare resource utilization costs were classified into two categories: drug costs and non‐drug costs. Drug costs were defined as expenses related to concomitant AA medications, while non‐drug costs included administration expenses associated with outpatient visits.

HCRU costs were estimated using anonymized claims data from JMDC Inc. The JMDC claims database has been collecting claims data from health insurance associations in Japan since January 2005. As of March 2025, the JMDC claims database includes data from over 23 million cumulative enrollees. The dataset comprises inpatient, outpatient, and pharmacy claims issued during healthcare visits [31, 32]. In the database analysis, the study period was from January 1, 2022, to November 30, 2024. Patients with AA were identified using the International Classification of Diseases 10th revision (ICD‐10) code L63 and relevant reimbursement codes in Japan, excluding cases with suspicious diagnoses. HCRU costs were estimated by calculating the average medical costs for each SALT score category (≤ 10, 11–20, 21–49, or ≥ 50). Patients assumed to be classified into the SALT score ≤ 10, SALT score ≥ 50 categories were predicted based on information related to diagnosis, treatment duration, and prescribed medications. The average medical costs were calculated for patients with AA who met the SALT score ≤ 10, SALT score ≥ 50, as well as for the overall population. The average medical costs for SALT score of 11–20 and SALT score of 21–49 were estimated via back‐calculation using a weighted average formula based on the patient distribution across SALT score categories.

Adverse event management costs were estimated by summing the products of the incidence rates of each adverse event and the treatment cost per patient. The types and doses of medications were determined based on expert opinions from four clinical experts in the treatment of AA. For cost estimation, the price of the most frequently prescribed generic drug in the claims data for patients with AA was used for medications with generic versions, and the price of the branded drug was used for medications without generic versions.

Indirect costs related to productivity loss were estimated using statistical data from the Japanese government and findings from a study in Japan that applied the work productivity and activity impairment questionnaire (WPAI) targeted to AA [25, 26, 33]. According to these data, indirect costs were estimated by calculating the loss of working hours associated with health states defined by SALT scores. The average age, sex distribution, and proportion of working‐age individuals (aged ≥ 18 years) in the target population were estimated using the JMDC claims database. The proportion of workers among those aged ≥ 18 years was calculated as per the assumption of retirement at 65 years of age [25, 26].

### 2.6 Sensitivity Analysis

A one‐way sensitivity analysis (OWSA) was conducted for the upper and lower bounds of each parameter used in the base‐case model to identify parameters that have strong associations with the results. The range of variation for each parameter in the OWSA was set as per the 95% confidence intervals reported in the referenced data. In cases where 95% confidence intervals were unavailable, the range of variation was set at ±20% of the values used in the base case.

In addition, a probabilistic sensitivity analysis (PSA) with 10 000 iterations was conducted. In each iteration, all parameters were assigned values randomly drawn from their respective statistical distributions, and the incremental cost‐effectiveness ratio (ICER) was calculated. Beta distributions were used for probabilities and utilities, Dirichlet distribution for SALT score, and gamma distributions for costs. Standard errors (SEs) were assumed as 10% of the base‐case values when fitting each statistical distribution.

### 2.7 Scenario Analysis

As a scenario analysis, an evaluation was conducted from the payer perspective. In this scenario, the analysis excluded indirect costs associated with productivity losses from the base‐case analysis.

## 3 Results

### 3.1 Base‐Case Analysis

The results of the base‐case analysis from a societal perspective are shown in Table 2. Compared with no treatment, ritlecitinib 50 mg yielded incremental QALYs of 1.09 QALYs and incremental costs of 5 262 471 JPY (34 766 USD). The ICER of ritlecitinib 50 mg versus no treatment was 4 816 589 JPY (31 820 USD) per QALY, less than the ICER of 5 000 000 JPY (33 032 USD) per QALY that is considered cost‐effective according to the threshold of the Health Technology Assessment of Japan.

**TABLE 2:** Result of base‐case analysis (societal perspective).

	Ritlecitinib 50 mg	No treatment	Difference
Total cost (JPY)	44 942 032	39 679 561	5 262 471
Drug acquisition cost	7 514 374	0	7 514 374
HCRU cost	5 727 112	6 005 173	−278 061
AE cost	3968	3918	50
Indirect cost	31 696 578	33 670 470	−1 973 892
Total QALY	17.67	16.58	1.09
ICER (JPY/QALY)	4 816 589/QALY		

Abbreviations: AE, adverse event; HCRU, healthcare resource utilization; ICER, incremental cost‐effectiveness ratio; JPY, Japanese yen; QALY, quality adjusted life year.

### 3.2 Sensitivity Analysis

**FIGURE 2:**
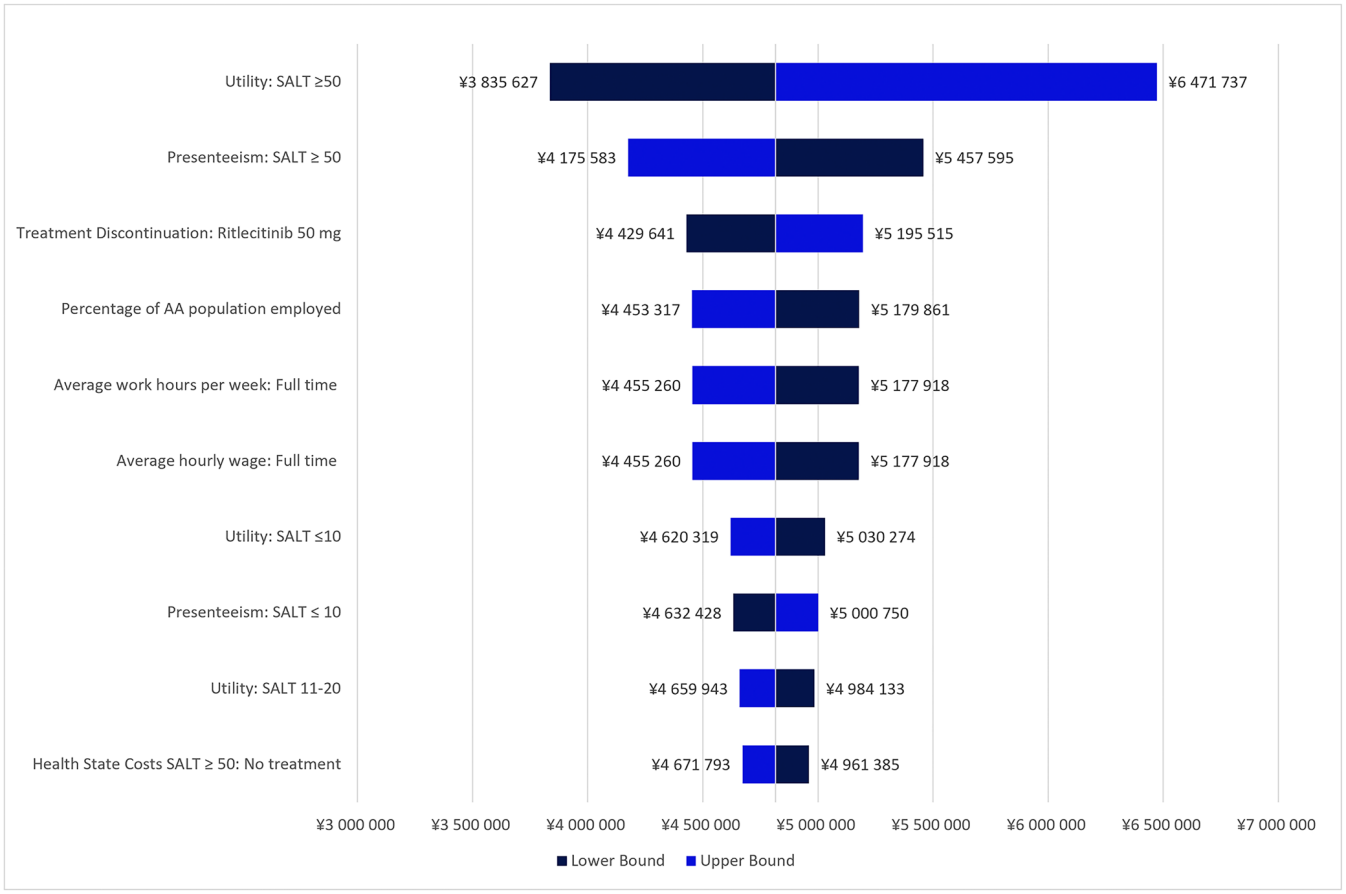
Tornado diagram for one‐way sensitivity analysis (societal perspective). SALT: Severity of Alopecia Tool.

The results of the OWSA are shown in Figure 2. The ICER varied from 3 835 627 JPY (25 339 USD) per QALY to 6 471 737 JPY (42 754 USD) per QALY. The parameter with the most notable effect on the ICER was the utility for patients with SALT score ≥ 50, followed by the percentage of work time missed due to presenteeism for patients with SALT score ≥ 50, and the discontinuation rate of ritlecitinib 50 mg.

**FIGURE 3:**
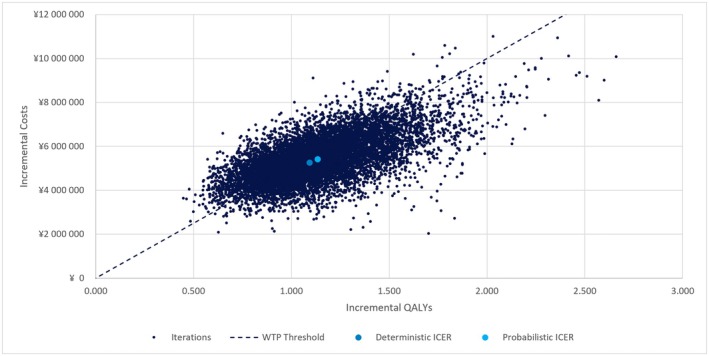
Cost‐effectiveness plane resulting from probabilistic sensitivity analysis (societal perspective). ICER: Incremental Cost‐Effectiveness Ratio, QALY: Quality Adjusted Life Year, WTP: Willingness‐To‐Pay.

**FIGURE 4:**
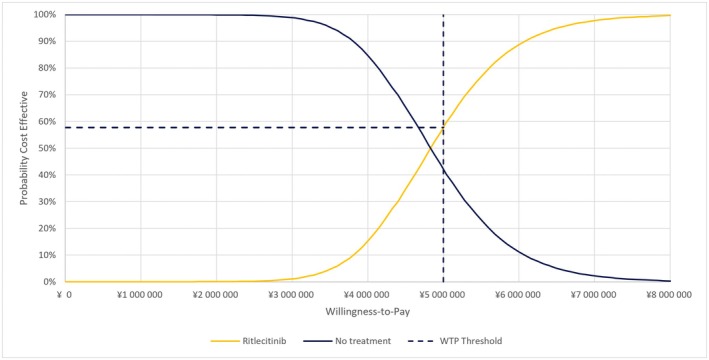
Cost‐effectiveness acceptability curve of ritlecitinib versus no treatment (societal perspective). WTP: Willingness‐To‐Pay.

In the PSA, the mean ICER was 4 771 815 JPY (31 524 USD) per QALY, which was consistent with the base‐case analysis (Figure 3). The cost‐effectiveness acceptability curve is shown in Figure 4. At a WTP threshold of 5 000 000 JPY (33 032 USD), the probability that ritlecitinib 50 mg is cost‐effective compared with no treatment was 57.7%.

### 3.3 Scenario Analysis

The results of the scenario analysis from the payer perspective are shown in Table 3. Compared with no treatment, ritlecitinib 50 mg yielded incremental QALYs of 1.09 and incremental costs of 7 236 363 JPY (47 806 USD). Accordingly, the ICER of ritlecitinib 50 mg versus no treatment was 6 623 236 JPY (43 755 USD) per QALY. The results of the OWSA are shown in Figure S1 and of the PSA in Figures S2 and S3.

**TABLE 3:** Result of scenario analysis (payer perspective).

	Ritlecitinib 50 mg	No treatment	Difference
Total cost (JPY)	13 245 454	6 009 091	7 236 363
Drug acquisition cost (JPY)	7 514 374	0	7 514 374
HCRU cost (JPY)	5 727 112	6 005 173	−278 061
AE cost (JPY)	3968	3918	50
Total QALY	17.67	16.58	1.09
ICER (JPY/QALY)	6 623 236/QALY		

Abbreviations: AE; adverse event; HCRU, healthcare resource utilization; ICER; incremental cost‐effectiveness ratio; JPY, Japanese yen; QALY, quality adjusted life year.

## 4 Discussion

In this cost‐effectiveness analysis from a societal perspective that included productivity loss, the ICER of ritlecitinib 50 mg was below the threshold in Japan of 5 000 000 JPY (33 032 USD) per QALY. This finding indicates that ritlecitinib 50 mg is more cost‐effective than no treatment for patients with AA involving ≥ 50% scalp hair loss and may help to characterize the clinical use of ritlecitinib in a real‐world setting in Japan.

Many cost‐effectiveness analyses are conducted from a payer perspective. However, guidelines and literature—including the International Society for Pharmacoeconomics and Outcomes Research Value Flower framework—have indicated that analyses from a societal perspective that include productivity loss are also required for cost‐effectiveness analyses [34, 35]. In the field of dermatology, skin diseases are known to lead to low productivity [13]. Productivity loss should therefore be included in the model to evaluate the cost‐effectiveness of the treatment for patients with skin disease. In this regard, several cost‐effectiveness analyses have been conducted (including productivity loss) in Japan [36, 37]. For example, a cost‐effectiveness analysis of abrocitinib for the treatment of patients with atopic dermatitis showed a similar trend, demonstrating that the cost‐effectiveness of abrocitinib 100 mg in combination with low‐ or medium‐potency topical corticosteroids was favorable compared with the standard of care (low‐ or medium‐potency topical corticosteroids alone) when productivity loss was considered [38]. In addition, an overseas study by Lekhavat et al. suggested that the use of diphenylcyclopropenone in the home is cost‐effective compared with that in the office for the treatment of patients with severe AA when considering direct and indirect costs [39]. According to these considerations, the base case in the present study adopted a societal perspective and estimated both the direct and indirect costs associated with the treatment of patients with AA.

The results of the OWSA showed that utility was the most influential factor affecting the ICER of ritlecitinib 50 mg for the treatment of patients with AA. When calculating utility, comprehensive measures such as the EuroQol 5 Dimensions (EQ‐5D) are commonly used [27, 40]. The EQ‐5D is based on responses to five indices (mobility, self‐care, usual activities, pain/discomfort, and anxiety/depression) and includes elements that may be unrelated to symptom improvement in patients with AA. In this regard, the National Institute for Health and Care Excellence (NICE) has stated that the EQ‐5D is often unsuitable for assessing improvements in HRQoL related to skin conditions [20, 41]. In addition, several recent studies have suggested that the EQ‐5D is insensitive for assessing HRQoL in patients with AA [42, 43]. The present analysis, therefore, applied the TTO method. The use of the TTO method has been discussed within the cost‐effectiveness evaluation system in Japan for ritlecitinib and has been adopted accordingly [44]. In future studies, determining which measures to use for HRQoL outcomes in cost‐effectiveness analyses of dermatological conditions will remain an important topic for consideration.

In the PSA, the probability that ritlecitinib 50 mg is cost‐effective compared with no treatment was 57.7% at a WTP threshold of 5 000 000 JPY (33 032 USD). There are no criteria for PSA to determine the probability of favorable cost‐effectiveness in the international guidelines. However, the result for PSA indicates the degree of uncertainty, with a 57.7% chance that the ICER will be lower than 5 000 000 JPY (33 032 USD) and a 42.3% chance that the ICER will be greater than 5 000 000 JPY (33 032 USD). Care is therefore required when interpreting these results regarding clinical practice.

This study has several limitations that should be considered when interpreting results. First, the 48‐week discontinuation rate of the ALLEGRO phase 2b/3 trial was used and extrapolated over the longer term for the present analysis. OWSA showed that the discontinuation rate of ritlecitinib 50 mg was a key parameter influencing the results. Since a lower discontinuation rate lowers the ICER, keeping it as low as possible is important when assessing the cost‐effectiveness of ritlecitinib for the treatment of patients with AA.

In our model, the discontinuation rate from ritlecitinib was set to 13.1% over 48 weeks. A systematic review from Spain has reported that the retention rate of baricitinib was approximately 50% at 4 years for patients with rheumatoid arthritis [45]. In contrast, the estimated retention rate for ritlecitinib in our model is approximately 30% (discontinuation rate is approximately 70%), which constitutes a conservative setting. Notably, however, the reported rates of retention or discontinuation differ between Japan and Spain, with Kaneko et al. reporting that the real‐world discontinuation rate from baricitinib for patients with rheumatoid arthritis in Japan was 30% within 12 months. More than half of these discontinuations were attributed to primary or secondary nonresponse [46]. Although there is a wide range of treatment options for patients with rheumatoid arthritis, options for those with AA remain limited, which should be considered when evaluating the discontinuation rates between these two patient populations.

In a patient preference study of patients with psoriasis in Japan, after long‐term efficacy, out‐of‐pocket cost was the second most important preference, followed by mode of administration and side effect [47]. Our analysis, however, did not consider the association of out‐of‐pocket costs with treatment discontinuation.

Second, because efficacy and safety were evaluated based on clinical trial results up to 48 weeks, the long‐term efficacy in the present study is an estimate. The ALLEGRO‐LT study is an ongoing, open‐label study investigating the safety and efficacy of ritlecitinib for up to 5 years, with results showing that the proportions of patients in the ritlecitinib 50 mg group achieving a SALT score ≤ 20 was 60.8% (observed data) or 46.1% (imputing data by LOCF [last observation carried forward]) and SALT score ≤ 10 was 50.8% (observed) or 37.7% (LOCF) at Month 24 [48]. In the present analysis, however, the estimated proportion of patients in the ritlecitinib 50 mg group at 2 years post‐initiation achieving a SALT score ≤ 20 was 41.2% and ≤ 10 was 29.8%. These results indicate a discrepancy between the present analysis at Month 24 and the ALLEGRO phase 2b/3 trial, which may arise from modeling the long‐term efficacy of ritlecitinib 50 mg using 48‐week data from the latter and, in turn potential underestimation. The guidelines for cost‐effectiveness evaluation in Japan [27] indicate that “The time horizon should be sufficiently long to evaluate the influence of the product on cost and effectiveness.” As with many other cost‐effectiveness analyses, the time horizon was set to lifetime in our model. However, long‐term estimates include any uncertainty, as several parameters, such as efficacy, safety, and discontinuation, were extrapolated using 48‐week data from the ALLEGRO phase 2b/3 trial.

Third, productivity loss was estimated using the human capital approach, which calculates productivity loss as “lost wages × hours of work absence.” This calculation has been criticized for potentially overestimating productivity loss in environments where unemployment exists (i.e., where there is a surplus labor force). Although there are also recommendations to use the friction cost method that includes only the period until a replacement worker is found, the present analysis study did not use this method given the difficulty of estimation. In addition, the human capital approach is also commonly applied in other cost‐effectiveness analyses and remains an area for future investigation.

Fourth, the present analysis only included a control group consisting of no treatment. In a clinical setting, although most patients with AA are eligible for ritlecitinib, a proportion may continue to receive ineffective treatments other than JAK inhibitors. Moreover, some patients with severe AA use a wig or receive topical immunotherapy, such as diphenylcyclopropenone (DPCP) or squaric acid dibutyl ester (SADBE), despite these treatments not being covered by national health insurance. To our knowledge, there are no clinical trials comparing JAK inhibitors with other active drugs in patients with AA who had an insufficient response to conventional treatments other than JAK inhibitors. We consider that the efficacy data from another clinical trial with a different patient background would increase uncertainty in our analysis. We therefore set the placebo arm of the ALLEGRO phase 2b/3 trial as the comparator and obtained clinical parameters from the ALLEGRO phase 2b/3 trial only. Both ineffective treatments other than JAK inhibitors and no treatment are considered suboptimal for patients with AA; however, receiving the former will incur higher costs than the latter. In the future, as evidence comparing ritlecitinib with active treatments accumulates, it is expected that cost‐effectiveness analyses will be conducted comparing ritlecitinib with active treatments in addition to no treatment.

Finally, there was a focus on data from Japan in the present analysis, given the aim to assess the cost‐effectiveness of ritlecitinib in this country. Notably, there are differences in healthcare systems between Japan and other countries, particularly regarding drug and treatment costs. Careful interpretation is therefore needed when applying these results to countries other than Japan.

In conclusion, this analysis demonstrated that ritlecitinib 50 mg is cost‐effective compared with no treatment in patients with AA involving ≥ 50% scalp hair loss and an insufficient response to existing treatments. The findings of this analysis may help inform the clinical use of ritlecitinib in a real‐world setting in Japan.

## Ethics Statement

The authors have nothing to report.

## Conflicts of Interest

The authors declare the following potential conflicts of interest with respect to the research, authorship, and/or publication of this article: A.Y., K.K., M.M., and K.N. are employees of Pfizer Japan Inc. S.S. and T.M. are employees of CRECON Medical Assessment Inc., which received funding from Pfizer Japan Inc. to undertake the research outlined in this study. S.K. and E.L. are employees of Pfizer Inc. R.U. reports advisory fees from Pfizer Japan Inc., and clinical trial fees not related to the submitted work from Pfizer Japan Inc. and Eli Lilly Japan K.K.

## Supporting information


**Data S1:** Supplementary Figures.

## Data Availability

The data sets generated during and/or analyzed during the current study are available from the corresponding author on reasonable request.
