# Supplementary material for: Cost‐Effectiveness Analysis of Ritlecitinib Compared With No Treatment in Patients With Severe Alopecia Areata in Japan
Source: J Dermatol. 2026 Mar 9;53(4):619–31. doi: 10.1111/1346-8138.70212 (PMC13075529; doi:10.1111/1346-8138.70212)
Supplement: Supplementary file 1 — Data S1: Supplementary Figures. [file JDE-53-619-s001.pdf]

Supporting information

Cost-effectiveness analysis of ritlecitinib compared with no treatment in patients with severe alopecia areata in Japan

Author names and affiliations

Akira Yuasa[1], Kazumasa Kamei[1], Shota Saito[2], Masashi Mikami[1], Tatsunori Murata[2], Samantha Kiyomi Kurosky[3], Ernest H Law[3], Kouki Nakamura[4], Rie Ueki[5]

1. Japan Access & Value, Pfizer Japan Inc., Tokyo, Japan
2. Health Economic Research Department, CRECON MEDICAL ASSESSMENT INC., Tokyo, Japan
3. HTA, Value & Evidence, Pfizer Inc., New York, NY, USA
4. Medical Affairs, Pfizer Japan Inc., Tokyo, Japan
5. Department of Dermatology, Juntendo Tokyo Koto Geriatric Medical Center, Tokyo, Japan.

Corresponding author

Akira Yuasa, Pfizer Japan Inc., Shinjuku Bunka Quint Building, 3-22-7, Yoyogi, Shibuya-ku, Tokyo 151-8589, Japan.

Email: akira.yuasa@pfizer.com

SUPPLEMENTARY FIGURES

SUPPLEMENTARY FIGURE 1.

Tornado diagram for one-way sensitivity analysis (payer perspective).

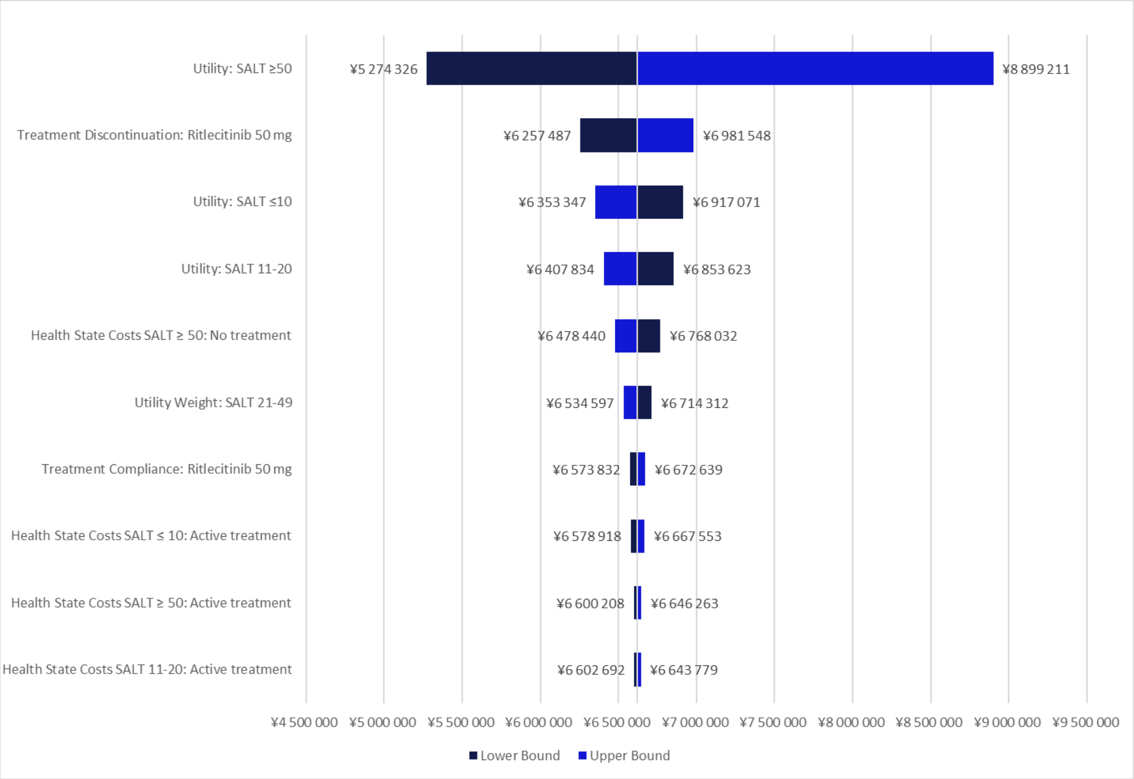

Abbreviations: SALT, Severity of Alopecia Tool.

SUPPLEMENTARY FIGURE 2.

Cost-effectiveness plane resulting from probabilistic sensitivity analysis (payer perspective).

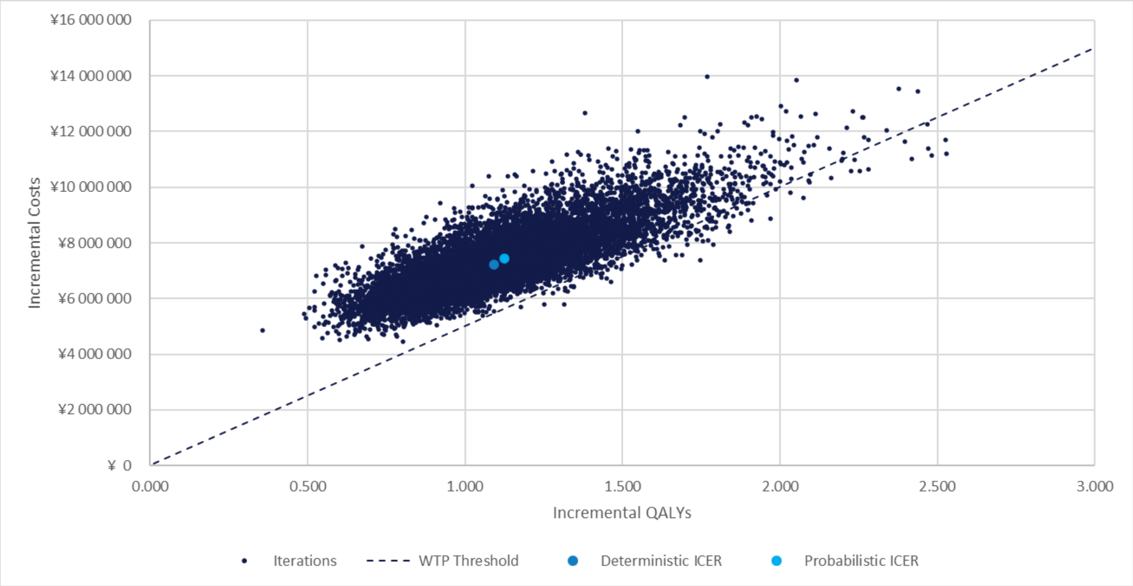

Abbreviations: ICER, incremental cost-effectiveness ratio; QALY, quality adjusted life year; WTP, willingness-to-pay.

SUPPLEMENTARY FIGURE 3.

Cost-effectiveness acceptability curve of ritlecitinib versus no treatment (payer perspective).

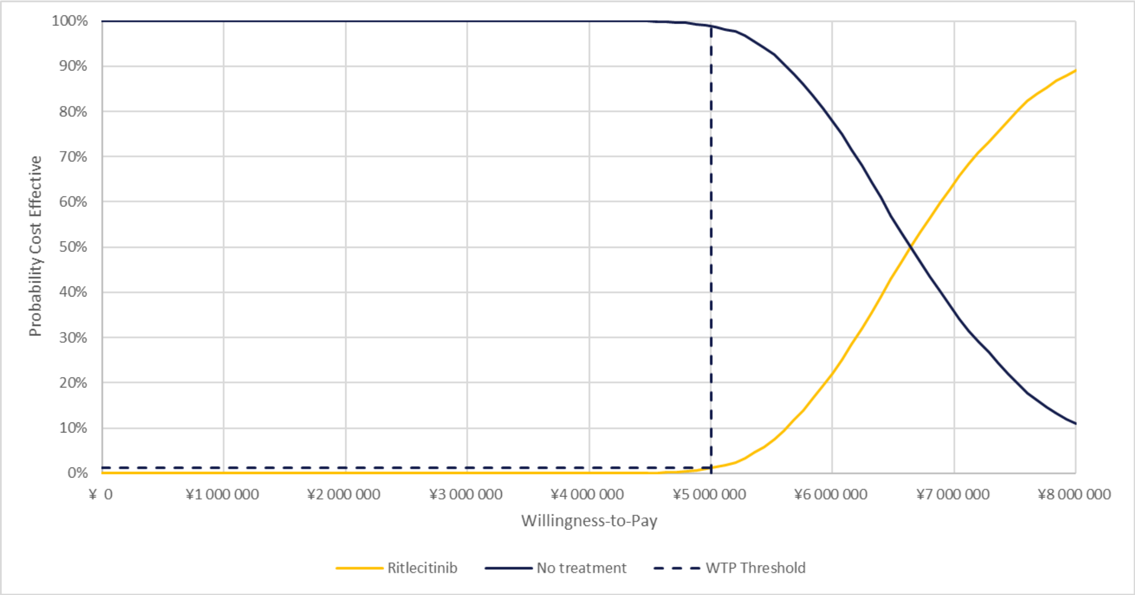

Abbreviations: WTP, willingness-to-pay.
